# Proton beam therapy for patients with distal cholangiocarcinoma: a multicenter prospective registry study (Influence of age factor)

**DOI:** 10.1093/jrr/rrag018

**Published:** 2026-04-20

**Authors:** Hideya Yamazaki, Kei Shibuya, Takuya Kimoto, Motohisa Suzuki, Masao Murakami, Kazuki Terashima, Takashi Iizumi, Masaru Wakatsuki, Takashi Ogino, Masaru Takagi, Masayuki Araya, Sae Matsumoto, Hiroyuki Ogino, Takayuki Hashimoto, Hidehiro Hojo, Nobuyoshi Fukumitsu, Takeshi Yanagi

**Affiliations:** Department of Radiology, Graduate School of Medical Science, Kyoto Prefectural University of Medicine, 465 Kajiicho Kawaramachi-Hirokoji, Kamigyo-ku, Kyoto 602-8566, Japan; Gunma University Heavy Ion Medical Center, 3-39-22 Showa-machi, Maebashi, Gunma, 371-8511, Japan; Department of Radiology, Graduate School of Medical Science, Kyoto Prefectural University of Medicine, 465 Kajiicho Kawaramachi-Hirokoji, Kamigyo-ku, Kyoto 602-8566, Japan; Department of Radiation Oncology, Southern TOHOKU Proton Therapy Center, 172 Yatsuyamada, Koriyama City, Fukushima Prefecture 963-8052, Japan; Department of Radiation Oncology, Southern TOHOKU Proton Therapy Center, 172 Yatsuyamada, Koriyama City, Fukushima Prefecture 963-8052, Japan; Department of Radiology, Hyogo Ion Beam Medical Center, 1-2-1 Koto, Shingu-cho, Tatsuno City, Hyogo 679-5165, Japan; Department of Radiation Oncology, Proton Medical Research Center, University of Tsukuba Hospital, 2-1-1, Amakubo, Tsukuba, 305-8576, Japan; QST Hospital, National Institutes for Quantum Science and Technology, 4-9-1 Anagawa, chiba-shi, Chiba 263-8555, Japan; Medipolis Proton Therapy and Research Center, Medipolis Medical Research Institute, 4423, Higashikata, Ibusuki, Kagoshima, 891-0304, Japan; Proton Therapy Center, Sapporo Teishinkai Hospital, 1-3-1, Kita 33-jo Higashi, Higashi-ku, Sapporo, Hokkaido, 065-0033, Japan; Proton Therapy Center, Aizawa Hospital, 2-5-1 Honjou Matsumoto-City, Nagano, 390-8510, Japan; Proton Therapy Center, Fukui Prefectural Hospital, 2-8-1 Yotsui, Fukui City, Fukui Prefecture, 910-8526, Japan; Department of Radiation Oncology, Nagoya Proton Therapy Center, Nagoya City University West Medical Center, 1-1-1, Nagoya, Japan; Global Center for Biomedical Science and Engineering, Faculty of Medicine, Hokkaido University, 5-chome, Kita 14-jo Nishi, Kita-ku, Sapporo, Hokkaido, 060-8648, Japan; Department of Radiation Oncology and Particle Therapy, National Cancer Center Hospital East, 6-5-1 Kashiwanoha, Kashiwa-shi, Chiba, 277-8577, Japan; Department of Radiation Oncology, Kobe Proton Center, 1-6-8 Minatojima Minamimachi, Chuo-ku, Kobe 650-0047, Japan; Department of Proton, Narita Memorial Proton Center, 134 Haneihonmachi, Toyohashi-shi, Aichi 441-8029, Japan

**Keywords:** proton beam therapy, distal cholangiocarcinoma, octogenarian

## Abstract

This study aimed to investigate the role of proton beam therapy (PT) in patients with unresectable distal cholangiocarcinoma (dCCA), with a special focus on elderly patients. We analyzed data from an updated multi-institutional prospective registry that included all Japanese proton beam facilities. The endpoints were local control (LC), progression-free survival (PFS), overall survival (OS) and toxicity. We included 36 patients with unresectable dCCA who were treated with PT at a median prescribed dose of 60 Gy (RBE) in 30 fractions. With a median follow-up of 12.4 months, the median survival time was 18.9 months, and the 1-year OS were 68.8%. The 1-year LC and PFS rates were 80.6 and 42.7%, respectively. The 1-year OS, LC and PFS rates for elderly patients (aged ≥80 years) were 65.6, 82.3 and 40.9%, respectively, showing no significant difference compared to younger patients (OS: 74.1%, *P* = 0.853; LC: 78.7%, *P* = 0.708; PFS: 45.5%, *P* = 0.654). However, four patients (11.1%) experienced grade ≥3 PT-related adverse reactions, exclusively among elderly patients (4/24 = 16.6% vs. 0/12 = 0% in younger patients, *P* = 0.278). These reactions were significantly higher with prescribed doses of equivalent 2-Gy fractions (EQD2)>60 Gy (3/6 = 50%) than with EQD2 ≤ 60 Gy (1/30 = 3.3%, *P* = 0.0104). PT showed good efficacy and acceptable toxicity for unresectable dCCA, even in patients aged ≥80 years, who showed outcomes similar to those of their younger counterparts. However, for elderly patients, a higher prescribed dose of EQD2 ≥60 Gy should be used with caution.

## INTRODUCTION

Cholangiocarcinoma (CCA) is a heterogeneous cancer that develops in the bile ducts. CCA is classified based on its localization as intrahepatic, perihilar or distal (dCCA) [1, 2], representing a significant subset that accounts for ~30% of all cases [1–3]. As with other CCA sites, surgical resection is currently considered the only potentially curative treatment approach [1–4]. For unresectable cases, the prognosis remains poor, and the National Comprehensive Cancer Network guidelines list radiotherapy (RT) and chemoradiotherapy (CRT) as potential options, along with systemic therapy and clinical trial participation [4]. Biliary tract cancer is a heterogeneous group of tumors, with distinct natural histories in the hilar, dCCA and gallbladder cancer, each requiring different optimal treatment approaches [1–4].

While 20th-century RT demonstrated a merit in improving tumor control and survival rates compared to best supportive care [5], the 21st century has seen the introduction of advanced RT techniques, such as stereotactic body RT (SBRT), intensity modulated radiotherapy (IMRT) and particle beam therapy [6, 7]. Among these, particle beam therapy offers a significant physical advantage over photon RT [8–12] due to its superior dose distribution using a spread-out Bragg peak. Leveraging this potential, Japan has established a national database (proton-net) compiling prospective data from all proton beam facilities [13]. This robust database has been valuable in providing evidence of the efficacy of proton beam therapy (PT), particularly for rare diseases such as dCCA. To our knowledge, most studies have dealt with dCCA as part of a mixed CCA population including ours [10–12], and few studies have reported outcomes of definitive RT for dCCA [7, 14]. Previous analyses reported on patients who underwent PT between May 2016 and June 2021 [10–12]. This time, we updated the collection period to March 2023 to gather more patient data. Therefore, this study aimed to investigate the efficacy and toxicity of definitive PT in patients with unresectable dCCA.

Furthermore, a critical consideration in modern oncology is the management of elderly patients, who often present with comorbidities and may not tolerate aggressive treatments as well as their younger counterparts. Although Japan is facing a rapidly aging society, data on RT for elderly dCCA patients are limited.

Therefore, a secondary objective was to specifically evaluate the impact of age on outcomes, focusing on patients aged ≥80 years to provide useful information for treatment decisions in this vulnerable population, as this cohort included an elderly population with a median age of 81 years.

## METHODS AND MATERIALS

### Patients

Patients who underwent PT at the 11 participating centers were registered in the database. Patients were eligible for the study if they had unresectable dCCA without distant metastasis, including those who refused surgery. It is mandated policy in Japanese proton institutions that all cases should be discussed at multidisciplinary conferences before PT is administered. Of the 60 patients initially registered, 24 were excluded for the following reasons: 2 previous or one planned surgery or 21 recurrent disease after surgery. Thirty-six patients with unresectable dCCA were included in this study (Table 1).

**Table 1:** Patients characteristics

Variables	Strata	Total	Age <80	Age ≥80	*P*-value
		(*n* = 36)	*n* = 12	*n* = 24	
		No. (%) or median [range]	No. (%) or median [range]	No. (%) or median [range]	
Age	Years	81 [58, 91]	73 [58, 78]	85 [80, 91]	
Gender	Female	9 (25.0)	2 (16.7)	7 (29.2)	0.685
	Male	27 (75.0)	10 (83.3)	17 (70.8)	
ECOG performance status	0	20 (55.6)	9 (75.0)	11 (45.8)	0.187
	1	13 (36.1)	2 (16.7)	11 (45.8)	
	2	3 ( 8.3)	1 ( 8.3)	2 ( 8.3)	
Liver function	Normal	5 (13.9)	1 (8.3)	4 (16.7)	0.542
	Child Pugh A	30 (83.3)	10 (83.3)	20 (83.3)	
	Child Pugh B	1 ( 2.8)	1 (8.3)	0 (0.0)	
Diagnosis	Pathological	30 (83.3)	9 (75.0)	21 (87.5)	0.378
	Imaging + tumor markers	6 (16.7)	3 (25.0)	3 (12.5)	
Tumor diameter	mm	24.50 [9.00, 77.00]	21.00 [9.00, 33.00]	27.50 [12.00, 77.00]	**0.047**
T category	1	13 (36.1)	3 (25.0)	10 (41.7)	0.114^a^
	2	8 (22.2)	5 (41.7)	3 (12.5)	
	3	12 (33.3)	3 (25.0)	9 (37.5)	
	4	1 (2.8)	1 ( 8.3)	0 ( 0.0)	
	Unknown	2 (5.6)	0 ( 0.0)	2 ( 8.3)	
N category	0	14 (38.9)	4 (33.3)	10 (41.7)	0.721^a^
	1	21 (58.3)	8 (66.7)	13 (54.2)	
	Unknown	1 ( 2.8)	0 ( 0.0)	1 ( 4.2)	
Stage	1	11 (30.6)	4 (33.3)	7 (29.2)	0.222
	2	21 (58.3)	6 (50.0)	15 (62.5)	
	3	2 (5.6)	0 ( 0.0)	2 ( 8.3)	
	4	2 (5.6)	2 (16.7)	0 ( 0.0)	
Chemotherapy	No	24 (66.7)	6 (50.0)	18 (75.0)	0.157
	Yes	12 (33.3)	6 (50.0)	6 (25.0)	
Adjuvant	Yes	3	1	2	
Neoadjuvant	Yes	4	2	2	
Concurrent	Yes	12	6	6	
Distance to GI tract	≥ 1 cm and <2 cm	2 (5.6)	1 ( 8.3)	1 ( 4.2)	0.253
	<1 cm	33 (91.7)	10 (83.3)	23 (95.8)	
	≥2 cm	1 ( 2.8)	1 ( 8.3)	0 ( 0.0)	
Prescribed dose	Gy (RBE)	60.00 [50.00, 72.60]	60.00 [52.00, 67.50]	60.00 [50.00, 72.60]	0.165
Fractions	Fractions	28 [22, 30]	30 [25, 30]	28 [22, 30]	0.099
Overall treatment time	Day	31.50 [25.00, 75.00]	30.00 [28.00, 44.00]	35.50 [25.00, 75.00]	0.141

^a^ *P*-values were calculated between age <80 and age ≥80. Bold values indicate statistical significance.

The treatment protocol was selected in accordance with the unified treatment policy of the Japanese Society for Radiation Oncology [15]. The treatment schedule was mainly determined by the tumor location and extent, as well as the distance between the tumor and gastrointestinal tract. The most frequently used schedules were 60 Gy (RBE)/30 fractions (*n* = 17), 50 Gy (RBE)/25 fractions (*n* = 6), 54 Gy (RBE)/27 fractions (*n* = 4) and 67.5 Gy (RBE)/ 25 fractions (*n* = 4) (Supplementary Table S1). The details of the treatment at each institution have been described in Supplementary Table S2 and elsewhere [9–12]. Passive scattering (broad beams) was employed in eight institutions, whereas pencil beam scanning techniques were used in three institutions. For respiratory gating, the AZ-733VI respiratory gating system (Anzai Medical Co., Tokyo, Japan) was used, and treatment was performed during exhalation phase (amplitude gating). ‘Three institutions have used gold markers and one institution used motion tracking systems for daily imaging guidance and motion management.’

The main systemic therapeutic agents were gemcitabine, TS-1, and a combination of cisplatin and gemcitabine. We analyzed overall survival (OS) as the primary endpoint and local control (LC) rate, locoregional control rate, progression-free survival (PFS) and toxicity as secondary endpoints. LC of the tumor was defined as the absence of recurrence at the originally irradiated site.

The national database protocol (proton-net) was reviewed and approved by the Hokkaido University Hospital Ethical Review Board for Life Sciences and Medical Research (approval number 016-0106). The study was approved by the Ethics Committee of each center, and written informed consent was obtained from all patients. This study was conducted in accordance with the principles of the Declaration of Helsinki.

The radiation dose was estimated using the biological effective dose (BED) and equivalent 2-Gy fractions (EQD2), with α/β = 10 (Supplementary Table S1).

Adverse events were classified according to the National Cancer Institute Common Terminology Criteria for Adverse Events, version 4.0 [16].

### Statistical analyses

The Easy R (EZR) statistical package was used for statistical analyses [17]. Percentages were analyzed using Fisher’s exact test for gender, Eastern Cooperative Oncology Group (ECOG) performance status, liver function, diagnosis methods, T category, N category, stage classification, use of chemotherapy, distance to the gastrointestinal tract according to age and PT-related adverse reactions according to prescribed doses and age. Mann–Whitney U-tests were used to compare age, tumor diameter, prescribed dose, fractions and overall treatment time according to age. The Kaplan–Meier method was used to analyze OS, LC and PFS, which were compared using the log-rank test. Multivariate analysis was not performed because of the small number of patients included in this study. The event time was determined from the start date of PT. The cutoff values were set at the median or mean values, if not specified. Statistical significance was set at *P* < 0.05.

## RESULTS

### Patient characteristics

A total of 36 patients underwent PT for non-metastatic dCCA between May 2016 and March 2023. The detailed patient, tumor and treatment characteristics are presented in Table 1. The median age of all patients was 81 years (range: 58–91 years), 75.0% of the patients were male and 91.7% had a good performance status (0–1). The median tumor diameter was 24.5 mm (range, 9–77 mm), and the median prescribed dose was 60 Gy (RBE) (range, 50–72.6 Gy) in 28 fractions (range, 22–30 fractions). A larger tumor diameter was noted in older patients aged ≥80 years (median 27.5 mm) than in younger counterparts (21 mm, *P* = 0.047, Table 1).

### Local control, progression-free survival and overall survival rate in total population

**Fig. 1:**
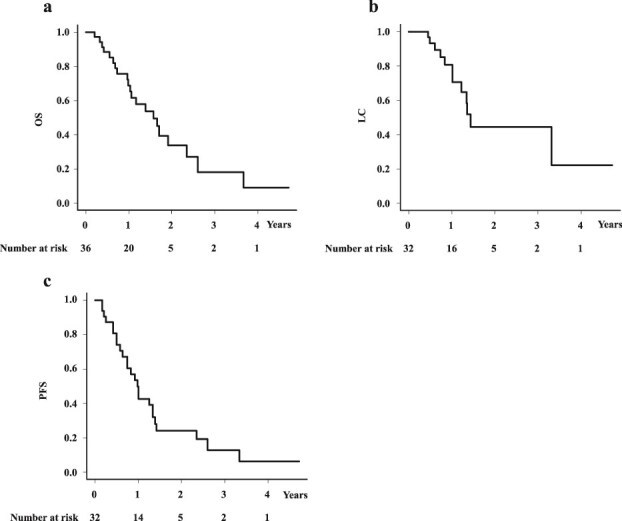
Overall survival rate, progression free survival rate and local control rates in total population. (a) OS rate. (b) LC rate. (c) PFS rate.

At the last follow-up, 15 patients were alive and 21 had died (13 death of disease, 2 other cause of death, 6 unknown cause of death). The median follow-up period was 12.4 months. The median survival time (MST) was 18.9 months (95% confidence interval [CI]: 11.8–28.1 months) for the total population, and the 1- and 2-year OS were 68.8% (95% CI: 49.6–81.9%) and 33.8% (95% CI: 15.8–53%), respectively (Fig. 1a). LC and PFS were analyzed in 32 patients because some patients could not receive follow-up examination to decide presence or absence of recurrence. LC was 80.6% (95% CI: 59.2–91.5%) at 1- year and 44.4% (95% CI: 20.8–65.7%) at 2 years (Fig. 1b). PFS was 42.7% (95% CI: 24.8–59.5%) at 1-year, and 24.0% (95% CI: 10.3–40.9%) at 2 years (Fig. 1c). Univariate analysis revealed no statistically significant predictors of OS, LC or PFS (Supplementary Table S3).

### Comparison between elder patients and younger counterpart

**Fig. 2:**
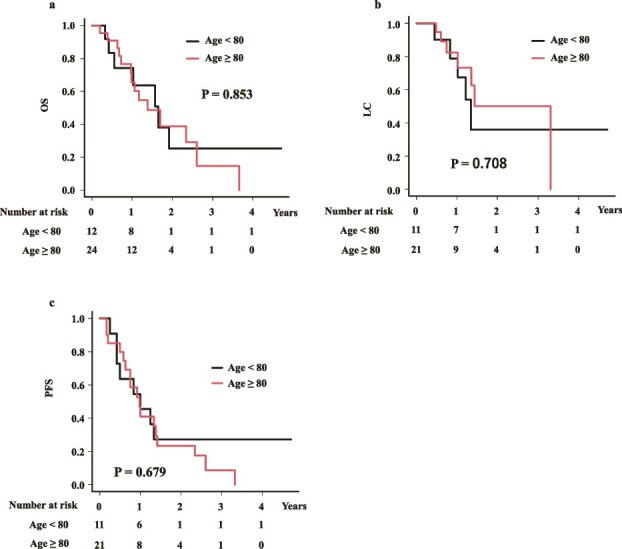
Comparison of outcomes between aged and younger counterpart. (a) OS rate. (b) LC rate. (c) PFS rate.

The MST for patients aged ≥80 years was 16.6 months (95% CI: 11.6–31.2 months), while for younger patients it was 19.8 months (95% CI: 4.9–not applicable months) (Fig. 2a). The 1- and 2-year OS rates were 65.6% (95% CI: 40.8–82.0%) and 38.9% (95% CI: 15.5–61.9%) for elderly patients and 74.1% (95% CI: 39.1–90.9%) and 25.4% (95% CI: 4.0–55.7%) in younger patients, showing no statistically significant difference (Fig. 2a). There was no statistically significant difference between the two groups (*P* = 0.853).

The 1- and 2-year LC were 82.3% (95% CI: 54.1–94.0%) and 50.2% (95% CI: 18.3–75.6%) for elderly patients and 78.7% (95% CI: 38.1–94.3%) and 36.0% (95% CI: 6.3–68.6%) for younger patients, respectively (Fig. 2b, *P* = 0.708).

The PFS at 1 and 2 years were 40.9% (95% CI: 19.0–61.8%) and 23.4% (95% CI: 7.4–44.4%) for elderly patients, and 45.5% (95% CI: 16.7–70.7%) and 27.3% (95% CI: 6.5–53.9%) for younger patients (Fig. 2c, *P* = 0.679), respectively.

### Proton beam therapy-related toxicity

The details of the toxicities related to PT are presented in Table 2. Four patients (11.1%) experienced adverse reactions of grade 3 (one acute toxicity occurred within 3 months after PT: 1/36 = 2.7%, three cases of late toxicity occurred after 3 months: 3/36 = 8.3%), all of which were diagnosed as PT-related toxicities occurred in elderly patients (4/24 = 16.6%, compared with 0/12 = 0% in younger patients, *P* = 0.278). Three were treated with a prescribed dose of EQD2 >60 Gy (3/6 = 50%), and one with an EQD2 ≤60 Gy (1/30 = 3.3%, *P* = 0.0104).

**Table 2:** Toxicity grade 3 or more^a^

Toxicity (*N* = 4)	Grade	Gender	Age	Schedule	Duration after PT (months)	Acute or late toxicity
Bile duct stenosis	3	F	82	54 Gy (RBE)/27 fr.	12	Late
Bile duct stenosis	3	M	90	67.5 Gy (RBE)/25 fr.	1	Acute
Cholangitis	3	M	84	64.8 Gy (RBE)/24 fr.	16	Late
Gastric bleeding	3	F	85	67.5 Gy (RBE)/25 fr.	8	Late

^a^fr. = fractions.

## DISCUSSION

This study aimed to evaluate the efficacy and toxicity of definitive PT in dCCA patients. To the best of our knowledge, this is the first report of a series of outcomes solely focusing on dCCA treated with definitive RT, including PT. PT has been shown to be effective, with a low incidence of severe toxicities. In particular, patients aged ≥80 years achieved outcomes similar to those of younger patients, despite having advanced tumors.

Although 20th-century RT was shown to improve tumor control and survival rates compared to best supportive care [5], dose escalation was difficult due to the proximity of bile duct malignancies to radiation-sensitive organs such as the bowel [18, 19]. Although intraluminal brachytherapy was used to increase the local dose, it did not consistently improve OS, particularly for larger tumors, and consequently its use has declined [20]. Consequently, the 21st century has seen the introduction of advanced RT modalities such as SBRT, IMRT and particle beam therapy, which offer promising avenues for dose escalation while minimizing normal tissue toxicity.

As many studies combine multiple sites of extrahepatic cholangiocarcinoma, there are limited data on definitive RT for dCCA (Table 3). The evolution of classification has complicated the assessment of treatment outcomes in patients with dCCA. Previously, a vertical classification of the bile duct was used in the 20th century, with the bile duct being categorized as hilar and proximal (upper), middle or lower (distal) [21]. Currently, the choice of surgical treatment varies significantly depending on whether the lesion is proximal or distal to the hepatic hilar region. Consequently, a clear distinction between ‘perihilar’ and ‘distal’ has been established and has become the standard practice [1–3]. The preferred surgical intervention for dCCA is pancreaticoduodenectomy, similar to that for pancreatic cancer. Therefore, several studies have reported on pancreatic cancer and dCCA together [22]. However, dCCA exhibits distinct and unique pathophysiological features compared to other periampullary neoplasms and should therefore be discussed separately [22]. Laughlin *et al.* reported 3- and 5-year OS were 16 and 0% in the definitive RT group with 36 dCCA and 3 hilar CCA cases [23]. Bisello *et al.* reported 18 months of MST and a 30% 2-year OS for dCCA in a multi-institutional data collection [14]. Elganainy *et al.* reported 27 months of MST for dCCA majorly using IMRT. They reported that escalated dose radiation therapy (defined as doses >50.4 Gy in 28 fractions [59.5 Gy BED]) to selective portions of the gross tumor volume may not benefit patients with unresectable extrahepatic cholangiocarcinoma [7]. We reported that a distance of ≥2 cm between the tumor and the gastrointestinal tract may allow for a higher prescribed dose and was significantly associated with OS, occurring in 26.3% of patients with intrahepatic cholangiocarcinoma and 15.3% with perihilar cholangiocarcinoma [12]. In this dCCA analysis, only one patient (2.7%) had a tumor-gastrointestinal tract distance ≥2 cm (data not shown). Based on the results of the above research, radiation dose escalation is especially difficult for dCCA because of the adjacent location of the gastrointestinal tract compared with other sites of bile duct cancer. The MST for dCCA has been reported as 18–27.1 months, with 2-year OS rates ranging from 30 to 69% (Table 3). For extrahepatic bile duct cancer in general, MST of 11–20 months and 2-year OS rates of 16–44% have been reported (Table 3). Our data (MST 18.9 months and 2-year OS 33.8%) were comparable to those of others. PT shows comparable outcomes to other modalities, such as IMRT and/or CRT, and a comparison with other modalities is warranted.

**Table 3:** Selected studies of radiotherapy for unresectable dCCA^a^

Authors	PY	Trial	RT	*N* (dCCA)	*N* (Total)	% of dCCA	MST (months) dCCA	MST (months) Total	2yOS (%) dCCA	2yOS (%) Total	Toxicity	
Ghafoori *et al.* [24]	2011	Retro	EBRT ± BT ± CT 45 (40–80) Gy /(1.8–3 Gy/fr.)	21	37	57%	NA	14	NA	22%	Acute Grade ≥3: 5 (14%)	21 distal, 16 proximal
Moureau-Zabotto [25]	2013	Retro	EBRT ± CT 48.25 (30–78 )Gy /(1.8–2Gy) fr.	6	24	25%	NA	11	NA	44% (1y)	Grade ≥3: RT16%, CRT37%	16 hilar, 2 intermediate, 6 distal
Autorino *et al.* [26]	2016	PII	EBRT ± BT ± CT 50.4 Gy/28 fr. ± BT:15–20 Gy	9	27	33%	NA	14	NA	27%	Acute GI Grade ≥3: 18.5%	18 proximal, 9 distal
Elganainy *et al.* [7]	2018	Retro	EBRT ± CT (35 3DCRT, 44 IMRT, 1 proton) 50.4 (30–75) Gy /28 fr.	18	80	23%	27.1	18.7	NA	33%^**^	Acute grade ≥3: 15%^***^ Acute GI grade ≥3: 11% Late grade ≥3: 28%	62 hilar,18 distal Dose escalation using IMRT/PT
Bisello *et al.* [14]	2019	Retro multi	EBRT ± CT ± BT 50(16–75) Gy/(1.8Gy/fr)	25^*^	76	NA	18	13.5	30%	26%	Acute GI and hematological Grade ≥3: 11% and 8.1%	25 distal, 51 other extrahepatic lesion
Laughlin *et al.* [23]	2022	Retro	16 3DCRT, 13 IMRT 50.4 (7.2–62.4)Gy	26	29	90%	NA	12.4^**^	NA	16%^**^	Two death during RT^****^ Biliary infection 17 (59%) Late GI Grade 3: 3 (10.3%)	3 hilar, 26 distal
Yamazaki *et al.* [27]	2022	Retro multi	Proton/carbon ± CT 70.2(44–77) Gy (RBE)/25(10–38) fr.	17	82	21%	28	20	69%	43%	Grade ≥3: Acute 2.2%, Late 2.7%	56 hilar, 17 distal, 9 GB
Current study	2026	Prospective data registry	proton ± CT 67.5 (50–72.6 ) Gy (RBE)/25 (22–30) fr.	36	36	100%	18.9	NA	34%	NA	Grade ≥3: Acute 2.7%, Late 8.3%	36 distal

^a*^No detail classification in EHCC (not specified as dCCA but not klastskin), ^**^read from figure, ^***^excluding-hematopoietic toxicity, ^****^one cholangitis, one rhabdomyolysis. BT = brachytherapy, CT = chemotherapy, 3DCRT = three dimensional conformal radiotherapy, EBRT = external beam radiotherapy, fr. = fractions, GI = gastrointestinal, IMRT = intensity modulated radiotherapy, NA = not available, PY = year of publication, PII = prospective phase II study, retro = retrospective, retro multi = retrospective multi-institutional data collection.

Grade ≥3 toxicity was observed in 16–37% of patients in the photon group and in 4.9–11.1% of patients in the PT group (Table 3). Specifically, acute toxicity grade ≥3 was 14–18.5% with photon therapy and 2.2–2.7% with proton therapy. Late toxicity grade ≥3 was 10.3–28% with photon therapy and 2.7–8.3% with proton therapy (Table 3). Although we could not compare the frequency of PT toxicity to that of photon therapy focusing on dCCA, PT showed a lower frequency of grade ≥3 toxicity than photon therapy.

The aging of the population has recently become a problem in cancer treatment in Japan, including for bile duct cancer. Although surgical procedures and systemic therapies are the preferred treatments for dCCA, they can be challenging for elderly patients, particularly those aged ≥80 years. Minimally invasive treatment is important for this group, and RT could be a good option for those who are not suitable for surgery. Notably, a dose of >60 Gy should be prescribed with caution for elderly patients, since escalating the dose did not improve efficacy and could increase toxicity, particularly in this elderly group aged ≥80, as observed in our cohort. We can speculate that the causes of increased and severe adverse events of PT in the elderly are age-related physiological decline and decreased reserve capacity, or the decreased ability of organs and tissues to regenerate. Therefore, 50–60 Gy may currently be the appropriate prescribed dose for dCCA.

The superiority of PT over photon RT has been demonstrated in recent paper showing improved OS rates for extrahepatic cholangiocarcinoma [28]. However, this superiority is not evident when comparing PT with photon RT using advanced RT techniques, such as SBRT, IMRT. Furthermore, dCCA is usually treated with surgery and/or systemic therapy whenever possible. Consequently, the number of cases treated with photon RT is small, and the patients are often elderly and/or in poor health. Therefore, data on photon RT for this specific indication is scarce, making comparison difficult. Since little data on PT for dCCA exists, this manuscript aims to present basic PT data to provide fundamental information for future comparisons. While survival improvement is mild and not evident, a reduction in adverse events appears promising and further exploration is warranted.

This study has several limitations. First, patient backgrounds were heterogeneous due to selection bias, despite the prospective accumulation of multicenter data. This heterogeneity arose because only dose fractionation was stipulated in the protocol, while irradiation margins, normal tissue dose constraints and follow-up methods were determined independently at each facility. Such variations may compromise the completeness of the data.

Second, although some patients received systemic therapeutic agents (e.g. gemcitabine, TS-1 and cisplatin) were administered to some patients, the specific details regarding their timing (concurrent vs. sequential), duration and types were not uniformly collected. Therefore, the data were too heterogeneous to allow for a robust analysis of their individual or combined impact with PT on OS, PFS, LC or toxicity. Consequently, the confounding effect of systemic therapy on the reported outcomes could not be fully elucidated and we could not identify a role for systemic therapy (Supplementary Table S3).

Despite these limitations, this prospective multicenter registry study is one of the largest analyses of definitive PT for dCCA, providing useful information for the treatment of dCCA, particularly in elderly patients.

In conclusion, PT showed good efficacy with acceptable toxicity for unresectable dCCA, even in patients age ≥80 years, who showed similar outcomes to their younger counterparts. However, for elderly patients, a higher prescribed dose of EQD2 ≥60 Gy should be used cautiously, as this subgroup showed a higher incidence of grade ≥3 adverse reactions.

## Supplementary Material

Distal_supplemental_Tables_2nd_revise_rrag018

## Data Availability

The datasets used and/or analysed during the current study available from the corresponding author on reasonable request.
